# Apoptotic mitochondria prime anti-tumour immunity

**DOI:** 10.1038/s41420-020-00335-6

**Published:** 2020-10-07

**Authors:** Kate McArthur, Benjamin T. Kile

**Affiliations:** 1 Department of Biochemistry and Molecular Biology, Biomedicine Discovery Institute, Monash University, Melbourne, VIC 3800 Australia; 2 Faculty of Health and Medical Sciences, The University of Adelaide, Adelaide, 5005 Australia

**Keywords:** Apoptosis, Mitophagy, Cancer immunotherapy

Immunogenic cell death (ICD)—the ability of a dying cell to activate an immune response—is attracting increasing interest as a biological phenomenon, and as a potential means to augment anti-tumour immunity. In a recent article, Yamazaki and colleagues explore the role of autophagy in limiting the immunogenicity of cell death following radiation therapy and, in-turn, the abscopal effect^1^.

Apoptosis, first described in 1972 by Kerr, Wyllie and Currie^2^, is the best characterised of all programmed cell death modalities. In contrast to its counterparts such as necroptosis and pyroptosis, apoptosis proceeds in an immunologically silent manner. Key to this silence is the function of a family of proteases called Caspases (*cy*steine-requiring *asp*artate-specific prote*ases*). Caspases accelerate cellular demolition and clearance. It is increasingly apparent that this activity functions to prevent ICD, both directly (e.g., nuclear fragmentation and the inhibition of protein-synthesis ensures dying cells do not secrete inflammatory cytokines, and the direct deactivation of danger-associated molecular patterns (DAMPs) prevents unwanted immune activation of neighbouring cells) and indirectly (e.g., loss of cellular adhesion, membrane blebbing and display of ‘eat-me' signals promote engulfment).

Caspases prevent ICD during apoptosis, but they are not actually required for the death of the cell. That function is fulfilled by BAK and BAX, two BCL-2 protein family members. Their activation and subsequent oligomerisation causes mitochondrial outer membrane permeabilization (MOMP), permanently damaging the mitochondria and allowing the release of cytochrome-*c* into the cytosol. Cytochrome-*c* is then able to form part of the apoptosome, which sets the caspase cascade into motion. Recently, it has been shown that not only does BAK/BAX-mediated MOMP permit the release of mitochondrial inter-membrane space proteins, but also causes extensive remodelling of the mitochondria and exposure of the inner mitochondrial membrane to the cytosol. This phenomenon, called mitochondrial herniation, allows the release of matrix components including mitochondrial DNA (mtDNA)^3,4^. Cytosolic mtDNA, much like an invading viral genome, is a potent agonist of the cell’s innate immune surveillance machinery; the cytosolic DNA sensor *C*yclic *G*MP-*A*MP *S*ynthase (cGAS) and its signalling counterpart *St*imulator of *In*terferon *G*enes (STING), which induce rapid Type I IFN secretion^5,6^. Although mtDNA release appears to be a routine event during apoptosis, thanks to rapid caspase-mediated dismantling of the cell, mtDNA-stimulated IFN secretion is only apparent when caspases are disabled.

**Fig. 1:**
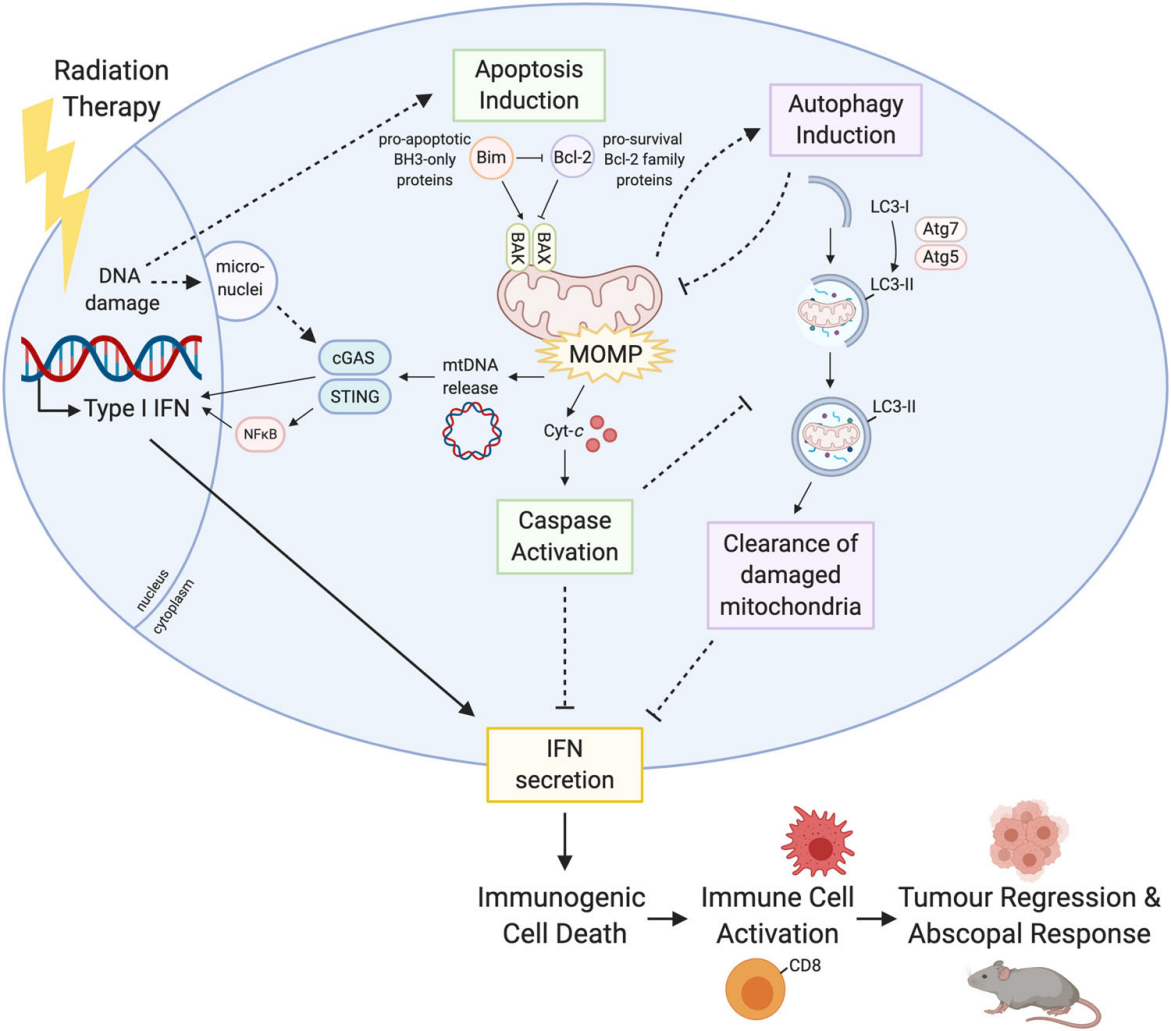
Summary schematic of the apoptotic and autophagic pathways induced by radiation therapy, and how this leads to immunogenic cell death (ICD). Figure created with BioRender.com.

Apoptotic MOMP has also been shown to trigger autophagy—a process by which cells deliver damaged or potentially toxic cellular components to lysosomes for degradation. Previous reports have shown apoptotic targeting of damaged mitochondria to lysosomes can reduce mtDNA-induced IFN secretion^7^. Thus, apoptotic mtDNA release, when given the opportunity (either by caspase inhibition or autophagy inhibition), is capable of transforming this normally silent cellular fate, into a form of immunogenic cell death (ICD) (Fig. 1).

In this work, Yamazaki and colleagues explore the response of breast cancer cells to radiation therapy (RT) under normal and autophagy-deficient conditions. Using both genetic and pharmacologic inhibition of autophagy, the authors found that autophagy-deficient mouse mammary carcinoma cells were not only more sensitive to RT, but produced more IFN in response to it. This was ablated in cGAS-deficient cells.

Previous reports have shown RT can stimulate the formation of micronuclei via DNA damage and genome instability, and that these micronuclei are recognised by cGAS to induce IFN and a pro-inflammatory response^8,9^. These reports also demonstrated that any abscopal responses to RT were also mediated via the cGAS-STING signalling axis. However, these studies differed from this work in one important area: the origin of the DNA recognised by cGAS.

Yamazaki and colleagues employed confocal microscopy to show RT induces the presence of cytosolic DNA foci which are in close proximity to mitochondria, appear to co-localise with mitochondrial transcription factor TFAM (but not with nuclear envelope protein Lamin B) and, most importantly, are absent in cells depleted of mtDNA (termed rho cells): suggesting that in the present model, it is mitochondrial, not genomic, DNA that signals IFN production. The authors demonstrated that RT was unable to induce IFN production in rho cells, and autophagy inhibition did not restore it. Overexpression of anti-apoptotic protein BCL-2, or loss of the pro-apoptotic protein BAX, significantly reduced the presence of cytosolic DNA species and the transcription of IFN following RT. Together, these findings strongly suggest BAK/BAX-mediated MOMP and mtDNA release are the drivers of IFN production in response to RT, and that autophagy plays a significant role in attenuating this IFN response in breast cancer cell lines.

Strikingly, when either control or autophagy-deficient cells were injected into the flanks of immunocompetent syngeneic mice (primary tumours), and control cells were injected into the opposing flanks (to model secondary lesions), RT directed at the primary tumours was only able to elicit abscopal responses at non-irradiated sites when the primary tumours were autophagy-deficient. Such responses were further enhanced when combined with CTLA4 inhibition.

These results mirror previous findings from the same group, in which mouse mammary carcinoma cells lacking Caspase-3 were shown to have increased IFN-production in response to RT, which also translated to enhanced abscopal responses (which again were further enhanced by CTLA4-blockade)^10^. Thus, despite subtle differences, both autophagy inhibition and caspase inhibition modulate apoptotic IFN production.

In a similar vein, a recent study by Giampazolias et al.^11^, showed that MOMP under caspase-deficient conditions, was able to induce potent anti-tumour effects. In this study, these effects were mtDNA-STING induced, signalled through both IFN-production and NFκB activation^11^. Indeed, there have been a few reports of pharmacological caspase inhibitors combining with cytotoxic treatments to increase anti-tumour responses^12,13^. More recently still, Han et al.^14^, reported Caspase-9 deficient tumours, but not their control-counterparts, demonstrated IFN-dependent CD8 T cell-mediated complete regression after RT, and impressively, mice remained protected from rechallenge 60 days later^14^.

On the face of it, caspases and autophagy appear to be playing similar roles in the context of apoptosis: shutting down the drivers of ICD, and in particular, mtDNA-mediated IFN production. However, several questions remain. First and foremost, why is an autophagic response still critical in caspase-proficient settings? Surely if caspase-mediated proteolysis is rapid and complete, the time-frame within which autophagy would have to clear damaged mitochondria would be too short? Are there ways of inducing MOMP but somehow slowing/decreasing caspase activation, and if so, how is this achieved? Are there differences in the way RT-induced cell death is regulated between different cell/tissue types? As discussed above, there is already evidence that this may be the case, with some groups reporting micronuclei formation and gDNA-cGAS activation, and this study clearly showing MOMP-induced mtDNA-cGAS activation.

A number of pre-clinical trials to establish the ability of autophagy inhibitors or caspase inhibitors to augment anti-cancer therapy are underway (clinicaltrials.gov). However, both drug classes currently come with a number of caveats: the only autophagy inhibitors approved for use are the quinoline derivatives Chloroquine and Hydroxychloroquine, which can have actions beyond autophagy; whilst caspase inhibitors often lack specificity between caspase family members, and generally possess only short half-lives. Encouragingly, more specific autophagy inhibitors (e.g., those targeting ATGB4 and ULK) are beginning to emerge^15^. Perhaps similar advances in caspase-inhibitors will occur. At a conceptual level, there is every reason to believe the approach holds genuine therapeutic potential. Based on the work of Yamazaki and colleagues, and the others discussed here, the prospect of killing dying cells in situ at the same time as manipulating them into driving anti-tumour immunity is very real.
